# Metabolic Regulation of Epithelial to Mesenchymal Transition: Implications for Endocrine Cancer

**DOI:** 10.3389/fendo.2019.00773

**Published:** 2019-11-26

**Authors:** Debasmita Bhattacharya, Anthony Scimè

**Affiliations:** Molecular, Cellular and Integrative Physiology Group, Faculty of Health, York University, Toronto, ON, Canada

**Keywords:** epithelial-mesenchymal transition, metabolism, endocrine cancers, mitochondria, metastasis

## Abstract

The last few decades have witnessed an outstanding advancement in our understanding of the hallmarks of endocrine cancers. This includes the epithelial to mesenchymal transition (EMT), a process that alters the morphology and functional characteristics of carcinoma cells. The mesenchymal stem cell like phenotype produced by EMT allows the dislocation of cancer cells from the primary tumor site with inheritance of motility, metastatic and invasive properties. A fundamental driver thought to initiate and propagate EMT is metabolic reprogramming that occur during these transitions. Though there remains a paucity of data regarding the alterations that occur during EMT in endocrine cancers, the contribution of deregulated metabolism is a prominent feature. This mini review focuses on metabolic reprogramming events that occur in cancer cells and in particular those of endocrine origin. It highlights the main metabolic reprogramming outcomes of EMT, encompassing glycolysis, mitochondria oxidative phosphorylation and function, glutamine and lipid metabolism. Comprehending the metabolic changes that occur during EMT will help formulate potential bioenergetic targets as therapies for endocrine cancer metastasis.

The preceding few decades have witnessed an outstanding advancement in our understanding of the hallmarks of cancer including the rare endocrine tumors. These include the signals that sustain their proliferative capacity, prevent apoptosis, enable replicative immortality, and induce angiogenesis (1). Another important consideration that has drawn significant attention is the epithelial to mesenchymal transition (EMT) of cancer cells that is thought to be required for their metastasis (2). The metastatic cascade of cancer cells involves loss of adhesion between cells, which results in their dissociation from the primary tumor and subsequently inheritance of a mesenchymal stem cell like phenotype. This is characterized by motility changes in cell to matrix interactions and plasticity to grow in different tissues (3). EMT results in tumor initiating cells (TICs) having properties similar to cancer stem cells (CSCs), which are associated with initiation, dissemination and recurrence of cancer (4). A fundamental driver thought to initiate and propagate EMT are metabolic alterations that occur during these transitions (5, 6). Though there is little information on the role of metabolism in EMT of endocrine cancers, an understanding in other cancers will provide some potential insight. This review will focus on how metabolic reorganization is an important regulator of EMT with particular regard to endocrine type cancers.

## Emt Induces TICs

EMT occurs when tumorigenic epithelial cells acquire a mesenchymal stem cell like phenotype by undergoing transcriptional, epigenetic, and metabolic changes. This causes loss of cell to cell adherence, imparting motility to the cells which are free to migrate and invade tissues, a process known as metastasis. Normally EMT is a physiological process necessary for organogenesis during embryonic development and for wound healing (7). However, it is thought that epithelial cancer cells also undergo EMT, becoming TICs, escaping from the primary tumor to enter in the blood circulation as potentially invasive cancer cells. EMT renders stem cell like properties to cancer cells, evidenced by increased self-renewal and survival, anchorage independent growth, and loss of differentiated characteristics (2).

TICs derived from EMT share similar characteristics with CSCs such as metastatic potential, chemo resistivity, anti-apoptotic function, gene expression signature, and metabolic profile (4). To sustain differences in morphology and function, the metabolic needs of EMT cells are different from the epithelial tumor cells from which they were derived, but similar to CSCs. Association between EMT and the acquisition of stem cell like properties have been observed in few endocrine cancers such as pancreatic, prostate, thyroid, and pituitary cancers (8, 9). Shaul et al., identified 44 common metabolic genes in CSCs which are also upregulated during EMT (10). Some of the important genes that are common between CSCs and cells that have undergone EMT are the glycan synthesis genes that modulate cell to cell interactions and gene expression (11, 12), lipid synthesis genes (13) and other genes associated with cancer aggressiveness, cell migration, and metastasis (14, 15). Some of these metabolic genes are also closely associated with EMT of the endocrine cancers.

**Figure 1:**
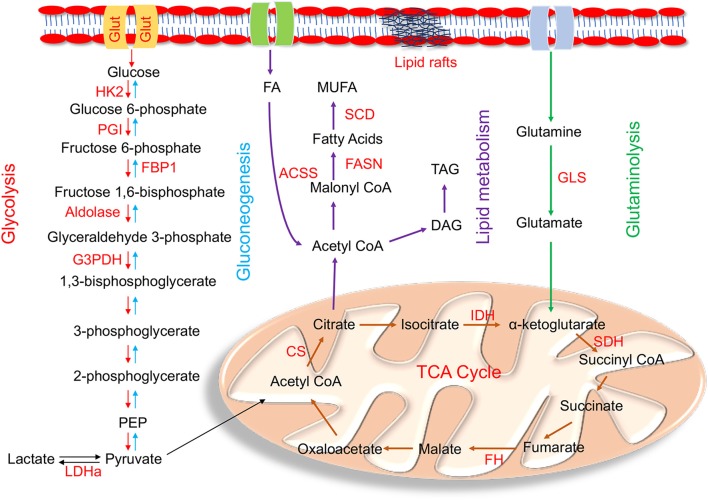
Enzymes within their pathways that are implicated in metabolic reprogramming during EMT. Schematic diagram of enzymes (highlighted in red) within the metabolic pathways of glycolysis, gluconeogenesis, mitochondrial, glutamine, and lipid metabolism alluded in the text that are altered during metabolic reprogramming that occurs in EMT. The main glycolytic enzymes that are upregulated in EMT are HK2 (hexokinase 2); PGI (phosphoglucoisomerase); aldolase, G3PDH (glyceraldehyde-3-phosphate dehydrogenase), and LDHa (lactate dehydrogenase a). FBP1 (fructose-1,6-bisphosphatase), the rate limiting enzyme of gluconeogenesis, is downregulated during EMT. Mutations in the tri-carboxylic acid (TCA) cycle enzymes linked to EMT are IDH (isocitrate dehydrogenase); SDH (succinate dehydrogenase); FH (fumarate hydratase); and CS (citrate synthase). The key enzyme involved in glutaminolysis is GLS (Glutaminase). *De novo* lipogenesis key enzymes involved in lipid metabolism are ACSS (acyl CoA synthetase); FASN (fatty acid synthase); and SCD (stearoyl CoA desaturase). Cells undergoing EMT also have high TAG (Triacylglycerols) levels.

How the metabolic network is reprogrammed to influence EMT remains unclear. The shift from epithelial to the mesenchymal morphology and loss of cell-cell adhesion is orchestrated by various transcription factor families (EMT-TFs) such as Snail, Twist and Zeb (16). EMT-TFs are known to regulate the transcription of various metabolic genes of different bioenergetic pathways (17). Glycolysis, mitochondrial oxidative phosphorylation (Oxphos), glutamine and lipid metabolism are the main energy producing pathways that maintain cellular harmony, but their metabolic deregulation and reprogramming influence initiation and progression of EMT and metastasis (Figure 1).

## Glycolytic Regulation of EMT

Unlike normal cells, cancer cells rely more on aerobic glycolysis, also known as the Warburg effect, to meet their elevated demand for energy during proliferation (18). In this regard, ATP is generated by a high rate of glycolysis followed by lactate production from pyruvate in the cytosol instead of pyruvate oxidation in the mitochondria, despite the presence of sufficient oxygen. The induction of genes associated with enhanced glycolytic flux also causes procurement of stem cell like properties in EMT (19–22). Indeed, the importance of aerobic glycolysis to EMT is characterized by the preponderance of deregulated glycolytic enzymes associated with cancer metastasis (23).

It is not clear how aerobic glycolysis favors EMT. One hypothesis is it provides a survival benefit against anoikis, which is a type of cell death that occurs when insufficient matrix attachment generates high levels of reactive oxygen species (ROS) to kill the cell (24). Normally anoikis would be a barrier to metastasis, but it is bypassed by EMT by critically decreasing oxidative metabolism via the Warburg effect to minimize production of ROS (25).

Despite the prominence of deregulated glycolysis in endocrine cancers there are few data available regarding its role in EMT. Proteomic analysis of endocrine pancreatic cells showed predominance of the Warburg effect and enhanced expression of factors involved in glucose metabolism (26). The potential implication for EMT is highlighted by metabolic profiling conducted on the exocrine pancreatic ductal adenocarcinoma (PDAC) that identified a subpopulation having a distinct glycolytic character. This subpopulation was strongly correlated with a stem cell like phenotype, indicative of EMT (27). Exposure of PDAC cell lines to known EMT inducers such as tumor necrosis factor-α and transforming growth factor-β resulted in conspicuous EMT accompanied by enhanced glycolysis and lactate secretion.

Dysregulation of glycolytic enzymes are evident in some endocrine cancers, but their role in EMT is unknown. For example, in pancreatic cancer cells, there is upregulation of the key enzymes of glycolytic metabolism and glucose transporters (28). Moreover, in different subsets of thyroid carcinoma, upregulation of hexokinase 2 (HK2), that phosphorylates glucose to form glucose 6-phosphate was observed (29). Intriguingly, in non-endocrine PDAC, HK2 is correlated with EMT and poor prognosis of the disease (30, 31). It has also been reported that breast cancer cells have augmented HK2 and its dose dependent inhibition by 2-deoxyglucose impede their EMT (32). Another glycolytic enzyme that is upregulated in many cancers is phosphoglucoisomerase (PGI). It mediates conversion of glucose 6-phosphate to fructose 6-phosphate and is associated with motility, migration, metastasis, and EMT in breast and lung cancers (33, 34). This is reflected by PGI mediated induction of EMT-TFs and increased metastatic potential in breast cancer cells (35). Although the role of PGI in EMT has been studied for many cancers, it is yet to be elucidated if it has a role in the endocrine tumor setting. Other glycolytic enzymes linked to metastasis and progression of endocrine type cancers are aldolase, glyceraldehyde-3-phosphate dehydrogenase and pyruvate kinase (36–38).

Also, some cancer cells can facilitate a metabolic shift toward aerobic glycolysis by upregulating glucose metabolism by impeding gluconeogenesis. For example, in several cancers including endocrine pancreatic cancer cells, loss of fructose-1,6-bisphosphatase (FBP1) that catalyzes the hydrolysis of fructose 1,6-bisphosphate to fructose 6-phosphate, is associated with increased cancer stem cell like phenotype and metastasis (39–42). FBP1 has been shown to be a direct target of Snail and Zeb1 transcriptional repression that promotes an increase for invasiveness of cancers cells (39, 43, 44). Restoring FBP1 expression, reduced glucose uptake, glycolysis and lactate generation concomitant with increased mitochondrial Oxphos that suppressed EMT (45).

In addition to the glycolytic enzymes, EMT induction occurs through enhanced activity and expression of glucose transporters, Glut1 and Glut3. They are important proteins that regulate glucose uptake, enabling rapidly dividing cells to sustain aerobic glycolysis (22). High levels of Glut1 are characteristic of endocrine cancers including thyroid carcinomas, pancreatic and high grade serous ovarian cancers where inhibition of Glut1 impeded glycolysis mediated cancer progression (26, 38, 46). Similarly, Glut3 expression in non-small cell lung cancer is associated with increased glucose uptake, activation of EMT-TFs and tumor cell invasiveness (47).

Another important factor that is positively regulated with cancer invasiveness and EMT is lactate dehydrogensase a (LDHa), a rate limiting enzyme converting pyruvate to lactate during aerobic glycolysis. Invasive pituitary adenomas exhibit high levels of LDHa both *in-vivo* and *in-vitro* (48). In intestinal-type gastric cancer cell lines, silencing LDHa downregulates Zeb2 and the synergistic decrease of LDHa and Zeb2 decreased cancer invasion, metastasis and poor prognosis (49). Also, in bladder cell lines, high levels of LDHa stimulated EMT leading to migration and invasion of the tumor cells (21) and silencing Ldha inhibited tumorogenecity in pancreatic cells *in vivo* (50). Similar to most aggressive cancers, LDHa was overexpressed in endocrine type cancers such as follicular thyroid carcinoma and papillary thyroid carcinomas compared to non-cancerous tissues (51).

## Mitochondrial Regulation of EMT

**Figure 2:**
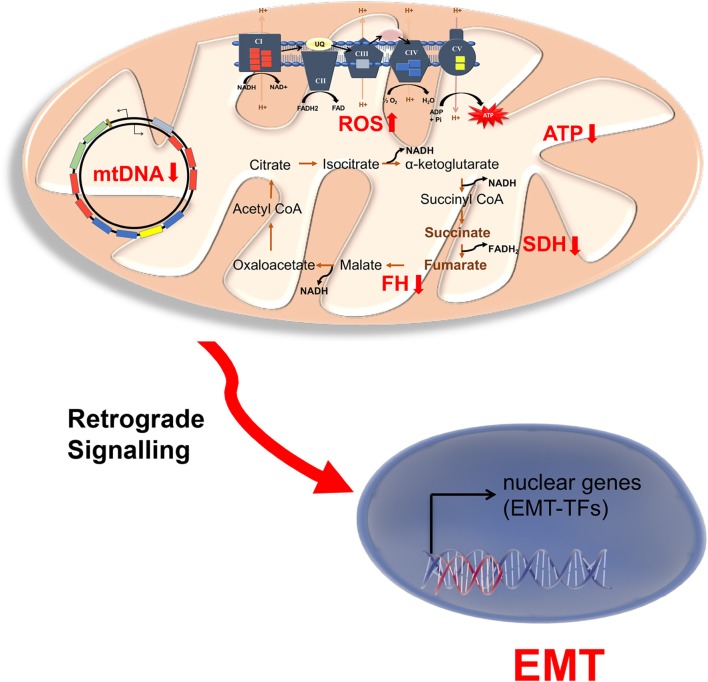
Mitochondrial dysfunction in endocrine cancers. Schematic representation of mitochondrial disruptions associated with EMT in endocrine cancers. NADH and FADH_2_ produced in the TCA cycle is utilized by electron transport chain (ETC) to produce ATP for Oxphos. As mitochondrial encoded genes are limiting for Oxphos, mutation in mitochondrial DNA (mtDNA) also causes low mitochondrial ATP and increased ROS (reactive oxygen species) production. The increase production of ROS and/or mutation in the TCA cycle enzymes succinate dehydrogenase (SCD) and fumarate hydratase (FH) result in accumulation of succinate and fumarate. These might be involved in retrograde signaling to activate nuclear EMT transcription factors, particularly in neuroendocrine cancers pheochromocytoma and paraganglioma.

Although there is a considerable amount of evidence linking mitochondrial dysfunction and cancer, the role of mitochondria in EMT has only recently been expounded (Figure 2). Decreased mitochondrial Oxphos capacity is usually associated with EMT of many cancer types and thus associated with promoting aerobic glycolysis. A combined RNAseq and metabolomics profiling of 20 different solid cancers has shown that downregulation of mitochondrial proteins, especially those involved in Oxphos, are associated with metastasis and EMT (52).

The reduction of Oxphos to enhance EMT and metastatic progression is also associated with mutant and/or reduced levels of mitochondrial DNA (mtDNA) (53). Importantly, four out of five electron transport chain (ETC) complexes are made up of functional subunits encoded from mtDNA that are indispensable for ATP production from Oxphos. For example, mutation of mtDNA encoding Complex I subunits of the ETC increases the propensity of oncocytic thyroid cancers (54). The importance of mtDNA mutations to metastasis is highlighted by experiments using cybrid technology, to distinguish the contribution of mitochondrial genome to cancer metastasis (55). Cybrids carrying mtDNA mutations resulted in higher metastasis compared to controls with no mtDNA mutation (55). Reduced mtDNA content might cause mitochondria to nuclear retrograde signaling, whereby the mitochondrial dysfunction triggers nuclei to express genes that activate EMT and metastasis (56, 57). ROS, a mitochondrial byproduct of the ETC, which can damage and mutate mtDNA, was also shown to induce metastasis of tumor cells (58). Intriguingly, decreased ETC complex I and III activity were associated with ROS production in the endocrine thyroid oncocytic carcinomas (38).

The EMT-TF Snail has been shown to reduce ETC complex formation by targeting cytochrome c oxidase (Cox), a Complex IV enzyme responsible for transferring electrons for mitochondrial respiration. Snail binds to the mtDNA promoter and downregulates the expression of three Cox subunits, COX6c, COX7a and COX7c, reducing the formation of Complex IV, thereby suppressing oxygen consumption and mitochondrial respiration (59).

A master regulator of mitochondrial biogenesis and enhancer of Oxphos, peroxisome proliferator-activated receptor gamma coactivator 1-alpha (Pgc-1α), has been shown to either suppress or activate EMT and metastasis depending on cancer types and metabolic cues of the tumor microenvironment (60). In endocrine thyroid cancers, Pgc-1α has been found to be downregulated concomitant with increased glycolytic flux (61). However, pancreatic CSCs have high levels of Pgc-1α compared to differentiated pancreatic tumor cells, suggesting that the association between Pgc-1α and EMT is dependent on stages and metabolic plasticity of cancer (62). Indeed, prostate cancers that are highly heterogeneous, provide different landscapes for Pgc-1α functioning. In some prostate cancers, Pgc-1α via an ERRα dependent mechanism increases the overall oxidative metabolism and blocks EMT and metastasis (63). On the contrary, androgen mediated AMPK activation causes prostate cancer cell growth through Pgc-1α mediated increase in mitochondrial biogenesis, glucose oxidation and fatty acid oxidation (64). Similarly, in non-endocrine breast cancer cells Pgc-1α mediated mitochondrial Oxphos and biogenesis facilitates metastasis (65). Silencing Pgc-1α impaired the invasion and metastasis without affecting the proliferation of the primary tumor.

One of the important metabolic pathways in mitochondria is the tri-carboxylic acid cycle (TCA) that provides reducing agents for Oxphos and metabolites for various biosynthetic pathways. Evident in endocrine cancers are mutations of TCA cycle operational enzymes, which are linked to EMT (66). The neuroendocrine tumors, pheochromocytoma and paragangliomas are associated with mutation of succinate dehydrogenase (SDH) that converts succinate to fumarate (66). In particular, a mutation of SDHb a subunit of SDH, is thought to alter glucose and glutamine utilization and cause epigenetic modifications that results in EMT (67–69). Moreover, SDH mutation is hypothesized to cause consumption of extracellular pyruvate to maintain the Warburg effect conducive for cell growth and thus EMT potential (70). This is through pyruvate carboxylation for aspartate biosynthesis, which utilizes glucose derived carbons produced in glycolysis. SDHb related changes in pheochromocytomas and paragangliomas are coupled to bioenergetic reprogramming where decrease in complex II of the ETC caused compensatory increase in Complexes I, III and IV with concomitant decrease in ATP levels (71). SDHb mutations cause upregulation of metastatic genes and epigenetic silencing of cell adhesion protein, keratin 19, leading to EMT and rendering the tumor cells more aggressive and invasive (67, 72).

Mutation of another important enzyme of the TCA cycle, fumarate hydratase (FH), which converts fumarate to malate, is also implicated in metastasis of pheochromocytoma and paraganglioma. This is due to accumulation of high levels of fumarate and succinate that act as oncometabolites by enhancing epigenetic modification of DNA hypermethylation, ROS production and changes in the mitochondrial structure (66, 73–76). In particular, fumarate accumulation has been shown to inhibit α-ketoglutarate-dependent dioxygenases that are involved in DNA and histone demethylation. Recently, fumarate buildup in renal cancers has been shown to cause EMT by inhibiting Tet dioxygenase mediated demethyation of antimetastatic miR-200 which is a known activator of metastasis and EMT (77).

In the TCA cycle, isocitrate dehydrogenase that converts isocitrate to α-ketoglutarate by decarboxylation, is also associated with many cancers (78). An oncometabolite formed due to mutation of isocitrate dehydrogenase, 2D hydroxyglutarate, induced metastasis and EMT in colorectal cancers by increasing Zeb1 expression (79). Finally, EMT is also correlated with citrate synthase (CS) activity, which converts oxaloacetate to citrate. CS induces morphological and metabolic alterations resembling EMT in human cervical carcinoma cells. It might also play a role in endocrine pancreatic cancers that are characterized by increased CS activity (80, 81).

Apart from glucose, many cancer cells also rely on glutamine as a nutrient source (82, 83). Glutamine metabolism replenishes the pool of TCA cycle intermediates (anapleuresis) that might be exported out of the mitochondria (cataploresis) for biosynthesis of building blocks. Thus, the use of glutamine for anapleuresis is very important for cancer cell proliferation and sustainability. Many carcinomas have an upregulation of the enzyme glutaminase 1 (GLS1), that catalyzes the first reaction of glutaminolysis, glutamine to glutarate for anapleuresis (84–86). In prostate cancer, loss of GLS1 activity was associated with decreased rate of glucose utilization and cancer progression (87). The potential importance of GLS to EMT in endocrine cancers, is highlighted by its inhibition that blocked EMT progression and metastasis by repressing Snail in non-endocrine colon and breast cancers (86). Recently drugs targeting glutaminase activity has been successfully used in preclinical trials to impede the invasiveness of tumor cells, thus counteracting EMT (85, 88, 89). Contrary to the role of GLS1, some studies have shown that over expression of GLS2, the mitochondrial isoform of GLS1, reduces tumor progression, invasion and poor prognosis as in human hepatocellular carcinoma tissues (90). It has been shown to cause repression of EMT through downregulation of Snail both *in vitro* and *in vivo* (91). NMR based metabolic profiling revealed that compared to sporadic tumors, pheochromocytoma and paraganglioma had higher levels of glutamine indicating the influence of glutamine metabolism in pathogenesis of these endocrine cancers (66, 92).

## Lipid Metabolism and EMT

In addition to aerobic glycolysis, *de novo* lipogenesis is augmented in many cancers, but little is known about the involvement of lipid metabolism in EMT. Lipidemic analysis on prostate cancer cells that had undergone EMT, showed increased triacylglycerols and fatty acid synthase (FASN) (93). FASN is an important enzyme in fatty acid synthesis catalyzing the NADPH-dependent condensation of acetyl-coenzyme A (CoA) and malonyl-CoA to produce palmitate. FASN, prominent in various cancer types, can increase expression of epidermal growth factor receptor (ErbB) that promotes EMT of breast cancer cells and invasive ductal carcinomas (94). High FASN expression levels in pancreatic cancer and papillary thyroid carcinoma patients is associated with poor survival rate, but its importance to EMT is unknown (95, 96).

Overexpression of acyl-CoA synthetases (ACSs), which convert long chain fatty acids into acyl CoA and stearoyl CoA desaturase-1 (SCD), induced EMT and increased cellular migration and invasion in colorectal cancer (97). Their dysregulation for EMT in endocrine cancers has not been studied. However, metabolic stress such as hypoxia or caloric restriction, enhances ACS expression which is involved in the growth of pancreatic cancer cells (98). SCD, the rate limiting enzyme converting saturated fatty acids into monounsaurated fatty acids maintains cellular homeostasis by regulating their ratio. Dysfunctional SCD results in high levels of monounsaturated fatty acids that is observed in several endocrine cancers. Also, it is considered as a predictive marker for metastasis and possible EMT (99). Importantly, inhibition of SCD in prostate cancer blocked tumor gowth and survival (100).

The plasma membrane (PM) integrity also has a crucial role in mediating EMT. This is evident by marked differences in the lipid composition of PM between normal epithelial cells and the cells that have undergone EMT (101). Cancer cells undergoing EMT might be influenced by a number of signaling pathways that are activated by extracellular ligands or receptors, which are attached to the PM. Recent evidence has shed light on reorganization of the PM that caused destabilization of lipid raft domains, which imparts motility and metastatic properties to the cancer cells undergoing EMT (102). Stabilization of the lipid rafts have been made possible by pharmaceutical and nutritional interventions that result in metastasis (103, 104). The importance of PM composition affecting EMT is also reinforced by the influence of cholesterol, whereby altering cholesterol content of plasma membrane is associated with increased mesenchymal stem cell like phenotype (102). Indeed, depletion of cholesterol content in mesenchymal like tumor cells by statin reduced PM fluidity, cell motility and metastatic potential (105).

## Conclusion and Perspectives

EMT causes dissociation of cancer cells from primary carcinomas, which migrate and disseminate to distant sites. In this review, we have summarized how cancer cell metabolic reprogramming reflected by changes in glycolysis, mitochondrial Oxphos, glutamine and lipid metabolism are involved in EMT. For many cancer types it is not known if one or more metabolic pathways are necessary for EMT and metastasis, nor if they operate independently or together within a metabolic framework. Moreover, it has not been resolved if the same metabolic reprogramming in different cancer types have opposite effects on EMT and metastasis. Although, endocrine cancers are one of the most aggressive cancers types, there is a gap in research connecting its metabolic deregulations and EMT. Elucidation of the compromised metabolic targets will help in identifying potential therapeutic targets.

Finally, recent advancement in the understanding of EMT, has unveiled that metastatic cascade is multifaceted, where EMT is required for tumor initiation and invasiveness, but mesenchymal to epithelial transition (MET) is crucial for the later stages of metastasis, particularly during metastatic colonization. Despite considerable understanding in metabolic regulation of EMT, there is not much known about metabolic control of MET. As MET occurs at a distant site from primary tumors and are functionally different than cells that have undergone EMT, it is likely that these cancer cells have a completely different metabolic reprogramming.

## Author Contributions

AS and DB both conceived and designed the idea and wrote the paper. DB and AS collected the information for the manuscript.

### Conflict of Interest

The authors declare that the research was conducted in the absence of any commercial or financial relationships that could be construed as a potential conflict of interest.
